# Thirteen‐year trends in risk scores predictive values for subsequent stroke in patients with acute ischemic event

**DOI:** 10.1002/brb3.2962

**Published:** 2023-03-28

**Authors:** Yunyun Xiong, Shang Wang, Zixiao Li, Marc Fisher, Liyuan Wang, Yong Jiang, Xinying Huang, Xing‐Quan Zhao, Xia Meng, Yongjun Wang

**Affiliations:** ^1^ Department of Neurology Beijing Tiantan Hospital, Capital Medical University Beijing China; ^2^ China National Clinical Research Center for Neurological Diseases Beijing Tiantan Hospital, Capital Medical University Beijing China; ^3^ Chinese Institute for Brain Research Beijing China; ^4^ Neurocardiology Center, Department of Neurology Beijing Tiantan Hospital, Capital Medical University Beijing China; ^5^ National Center for Healthcare Quality Management in Neurological Diseases Beijing Tiantan Hospital, Capital Medical University Beijing China; ^6^ Department of Neurology, Stroke Division, Beth Israel Deaconess Medical Center Harvard Medical School Boston Massachusetts USA; ^7^ Advanced Innovation Center for Human Brain Protection Capital Medical University Beijing China

**Keywords:** recurrence, scale, stroke

## Abstract

**Introduction:**

A high residual risk of subsequent stroke suggested that the predictive ability of Stroke Prognosis Instrument‐II (SPI‐II) and Essen Stroke Risk Score (ESRS) may have changed over the years.

**Aim:**

To explore the predictive values of the SPI‐II and ESRS for 1‐year subsequent stroke risk in a pooled analysis of three consecutive national cohorts in China over 13 years.

**Results:**

In the China National Stroke Registries (CNSRs), 10.7% (5297/50,374) of the patients had a subsequent stroke within 1 year; area under the curve (AUC) of SPI‐II and ESRS was .60 (95% confidence interval [CI]: .59–.61) and .58 (95% CI: .57–.59), respectively. For SPI‐II, the AUC was .60 (95% CI: .59–.62) in CNSR‐I, .60 (95% CI: .59–.62) in CNSR‐II, and .58 (95% CI: .56–.59) in CNSR‐III over the past 13 years. The declining trend was also found in ESRS scale (CNSR‐I: .60 [95% CI: .59–.61]; CNSR‐II: .60 [95% CI: .59–.62]; and CNSR‐III: .56 [95% CI: .55–.58]).

**Conclusions:**

The predictive power of the traditional risk scores SPI‐II and ESRS was limited and gradually decreased over the past 13 years, thus the scales may not be useful for current clinical practice. Further derivation of risk scales with additional imaging features and biomarkers may be warranted.

## Graphical abstract

The predictive power of the traditional risk scores SPI‐II and ESRS was limited and showed a gradual declining trend over the past 13 years.

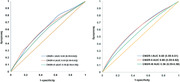

## 1 INTRODUCTION

The 2019 Global Burden of Disease Study found that stroke is the second leading cause of mortality worldwide (Tsao et al., 2022). The global age‐standardized prevalence rate for stroke was 2.24% (Tsao et al., 2022), while the weighted prevalence of stroke was 2.58% in China in 2019 (Tu et al., 2022). The rate of subsequent stroke ranged from 7.0% to 20.6% in patients with acute ischemic stroke (AIS) or transient ischemic attack (TIA) (Lin et al., 2021; Mohan et al., 2011). Subsequent stroke leads to an unfavorable functional outcome and has a serious impact on the quality of life of patients compared with those who only have a single ischemic stroke (Jørgensen et al., 1997).

Risk stratification of stroke recurrence is essential and a number of risk scores have been developed. The Stroke Prognosis Instrument‐II (SPI‐II) and the Essen Stroke Risk Score (ESRS) are two widely utilized models for long‐term risk prediction (Mohan et al., 2011), which mainly include traditional cardiocerebrovascular risk factors (age, smoking status, hypertension, diabetes, coronary heart disease, congestive heart failure, and prior stroke). Over the past 20 years since the ESRS and SPI‐II models were developed, the incidence and recurrence rates of stroke have declined as a result of new treatments and risk factor modification that emphasize secondary prevention (Carandang et al., 2006; Koton et al., 2020; Menon et al., 2015; Yeo & Yau, 2019). It is worth noting that some patients who have received the guideline recommended prevention measures still have a high residual risk of subsequent stroke (Ji et al., 2013; Ridker, 2016). This suggests that in addition to the common cardiocerebrovascular disease risk factors included in the stroke risk prediction models, there are other important factors that influence residual risk (Pan et al., 2021). Thus, the predictive ability of risk models for subsequent stroke may have changed over the past two decades. More importantly, there are few studies investigating the trends in the predictive power of risk models over the years.

We performed a pooled analysis of three China National Stroke Registries (CNSRs), including CNSR‐I, CNSR‐II, and CNSR‐III, to explore the 13‐year trends in risk prediction scores (ESRS and SPI‐II) for 1‐year subsequent stroke.

## 2 METHODS

### 2.1 Study design

**FIGURE 1:**
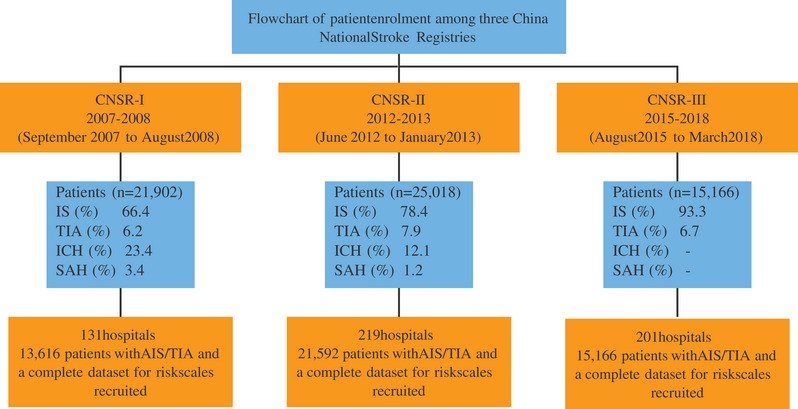
Flow diagram of participants in this study.

The CNSRs are a series of nationwide prospective registry studies, which had similar designs but were conducted independently at different time periods over 13 years. Details on the study design, rationale, and baseline data of CNSR‐I, CNSR‐II, CNSR‐III have been published previously (Li et al., 2016; Wang et al., 2011, 2019) and were summarized in Table S1. In brief, CNSR‐I, CNSR‐II, and CNSR‐III prospectively recruited consecutive patients with acute cerebrovascular events including individuals aged ≥18 years in China. The CNSR‐I was conducted from 2007 to 2008 and was designed to evaluate stroke care delivery and help to develop secondary stroke prevention strategies, and the CNSR‐II assessed the change in the quality of stroke care by comparing the results to CNSR‐I. CNSR‐III focused on identifying imaging and biological markers for the prognosis of ischemic events and was conducted from 2015 to 2018. In order to evaluate the trends of risk scores’ predictive values for subsequent stroke over 13 years, we performed a pooled analysis of CNSR‐I, CNSR‐II, and CNSR‐III. Patients with AIS or TIA and a complete data set for risk scales were enrolled in this study. Figure 1 shows the flow chart of participants in this study.

Information including demographic characteristics, medical history, and the National Institutes of Health Stroke Scale (NIHSS) scores at admission was extracted. The outcome of interest was subsequent stroke within 1 year including ischemic stroke, intracerebral hemorrhage, and subarachnoid hemorrhage. Patients were contacted over the telephone by trained research coordinators at 6 months and 1 year. Confirmation of cerebrovascular events was sought from the treating hospital, and suspected subsequent cerebrovascular events without hospitalization were judged by independent endpoint judgment committee. Each case fatality was either confirmed on a death certificate from the attended hospital or the local citizen registry.

The CNSR‐I, CNSR‐II, and CNSR‐III studies were approved by the Central Institutional Review Board in Beijing Tiantan Hospital (IRB approval number for the CNSR‐II study: KY2012‐005‐01; IRB approval number for the CNSR‐III study: KY2015‐001‐01), and written informed consents were obtained from all participants or their legally authorized proxies.

### 2.2 Risk scores

The parameters of SPI‐II and ESRS and points assigned to each parameter are detailed in Table S2. SPI‐II consisted of seven factors: age > 70 years (2 points), diabetes mellitus (3 points), hypertension (1 point), coronary artery disease (1 point), distinction between stroke and TIA at baseline event (2 points), congestive heart failure (3 points), and prior stroke (3 points). Low‐, medium‐, and high‐risk groups comprised patients with 0–3, 4–7, and 8–15 points, respectively (Kernan et al., 2000). The ESRS is a sum score calculated as follows (0–9 points): 2 points for age > 75 years and 1 point each for age 65–75 years, hypertension, diabetes mellitus, previous myocardial infarction, other cardiovascular diseases (except myocardial infarction and atrial fibrillation), peripheral arterial disease, smoking, and previous TIA or ischemic stroke in addition to the qualifying event. ESRS showed an annual risk of subsequent stroke of >4% in patients with an ESRS > 2 points (Diener et al., 2005).

### 2.3 Statistical analysis

Data are presented as mean with standard deviation (SD) or median with interquartile range (IQR) for continuous variables, and percentages were used for categorical parameters. Baseline characteristics were analyzed by chi‐square tests for categorical variables and analysis of variance or Kruskal–Wallis test for continuous variables. Between‐group differences in baseline characteristics were compared using absolute standardized difference, with >10% as statistical significance. We calculated the area under the receiver operating characteristic (ROC) to evaluate the predictive values of SPI‐II and ESRS for 1‐year subsequent stroke in all enrolled patients and in CNSR‐I, CNSR‐II, and CNSR‐III separately. A *p*‐values of <.05 was considered statistically significant. The analysis was conducted in SAS 9.4 (SAS, Cary, NC, USA) and R x64 4.1.0.

## 3 RESULTS

### 3.1 Clinical characteristics

A total of 50,374 patients with AIS or TIA were included in the analysis (CNSR‐I: 13,616; CNSR‐II: 21,592; CNSR‐III: 15,166). Demographics, vascular risk factors, NIHSS scores at baseline, and the proportions of patients with subsequent stroke at 1‐year among CNSR‐I, CNSR‐II, and CNSR‐III are listed in Table 1. The CNSR‐I has the eldest population, the lowest proportion of other cardiovascular diseases (except myocardial infarction and atrial fibrillation), and NIHSS ≤4. CNSR‐II has the highest smoking ever rate and proportion of peripheral artery disease. CNSR‐III has the lowest prior stroke or TIA rate. These three registries varied from each other, which may be representative of a broad spectrum of stroke patients in China.

**TABLE 1:** The clinical characteristics of CNSR series registries

	All	CNSR‐I	CNSR‐II	CNSR‐III			
Variables	(*N* = 50,374)	(*N* = 13,616)	(*N* = 21,592)	(*N* = 15,166)	Absolute standardized difference^a^ (%)	Absolute standardized difference^b^ (%)	Absolute standardized difference^c^ (%)
Age, mean ± SD	64.0 ± 1.9	65.3 ± 12.3	64.7 ± 12.0	62.2 ± 11.3	4.9	26.2	21.4
Men	32,388 (64.3)	8381 (61.6)	13,643 (63.2)	10,364 (68.3)	2.7	11.6	8.8
Smoking ever	17,879 (35.5)	3611 (26.5)	9516 (44.1)	4752 (31.3)	31.2	8.6	22.0
Diabetes mellitus	10,830 (21.5)	2870 (21.1)	4403 (20.4)	3557 (23.5)	1.4	4.7	6.1
Hypertension	32,131 (63.8)	8616 (63.3)	13,861 (64.2)	9654 (63.7)	1.5	0.7	0.8
Dyslipidemia	6666 (13.2)	1552 (11.4)	2657 (12.3)	2457 (16.2)	2.3	11.1	9.0
Coronary heart disease	6489 (12.9)	1961 (14.4)	2920 (13.5)	1608 (10.6)	2.1	9.6	7.4
Congestive heart failure	533 (1.1)	266 (2.0)	173 (0.8)	94 (0.6)	7.8	11.2	2.0
Previous myocardial infarction	1498 (3.0)	575 (4.2)	523 (2.4)	400 (2.6)	7.9	7.5	1.0
Other cardiovascular diseases (except myocardial infarction and atrial fibrillation)	5054 (10.0)	449 (3.3)	2506 (11.6)	2099 (13.8)	28.8	28.6	5.3
Peripheral artery disease	1095 (2.2)	84 (0.6)	893 (4.1)	118 (0.8)	21.9	1.9	19.9
Atrial fibrillation	3446 (6.8)	960 (7.1)	1467 (6.8)	1019 (6.7)	1.0	1.3	0.3
Prior stroke	14,896 (29.6)	4570 (33.6)	6971 (32.3)	3355 (22.1)	2.3	21.6	19.2
TIA	2283 (4.5)	698 (5.1)	1169 (5.4)	416 (2.7)	1.1	10.7	11.9
NIHSS ≤4	30,963 (61.5)	7317 (53.7)	13,731 (63.6)	9915 (65.4)	16.4	19.8	3.1

Abbreviations: CNSR, China National Stroke Registry; IQR, interquartile range; NIHSS, National Institutes of Health Stroke Scale; SD, standard deviation; TIA, transient ischemic attack.

^a^ Absolute standardized differences: Between CNSR‐I and CNSR‐II.

^b^ Absolute standardized differences: Between CNSR‐I and CNSR‐III.

^c^ Absolute standardized differences: Between CNSR‐II and CNSR‐III.

### 3.2 Subsequent stroke

During the 1‐year follow‐up period, 5297 (10.7%) patients had a subsequent stroke from the pooled data of CNSR‐I, CNSR‐II, and CNSR‐III; the rate of subsequent stroke in CNSR‐I was the highest (CNSR‐I 17.8% [2251/13,616] vs. CNSR‐II 7.3% [1573/21,592] vs. CNSR‐III 9.7% [1473/15,166]; *p* < .001).

### 3.3 Diagnostic values of SPI‐II and ESRS for 1‐year subsequent stroke

**FIGURE 2:**
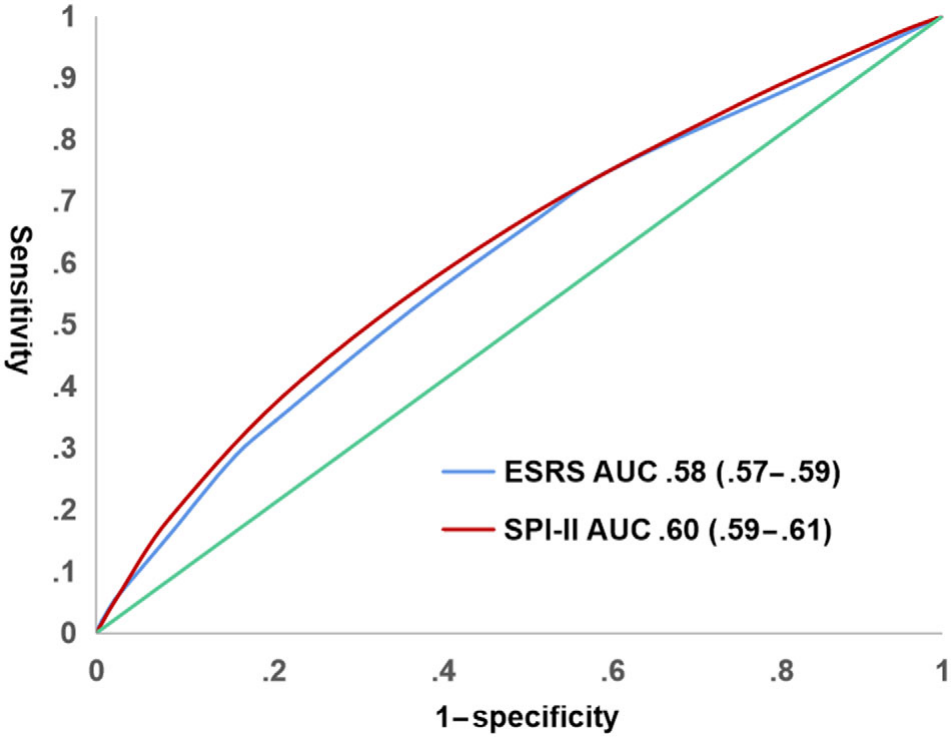
The diagnostic values of SPI‐II and ESRS for 1‐year subsequent stroke in the pooled analysis of CNSR‐I, CNSR‐II, and CNSR‐III. SPI‐II, Stroke Prognosis Instrument‐II; ESRS, Essen Stroke Risk Score; CNSR, China National Stroke Registry.

Figure 2 displays the diagnostic values for the pooled rates of subsequent stroke at 1‐year across CNSR‐I, CNSR‐II, and CNSR‐III for SPI‐II and ESRS. The values for area under the curve (AUC) of SPI‐II and ESRS were .60 (95% confidence interval [CI]: .59–.61) and .58 (95% CI: .57–.59), respectively. The SPI‐II had a better prediction performance in relation to subsequent stroke than the ESRS (*p* < .001).

**FIGURE 3:**
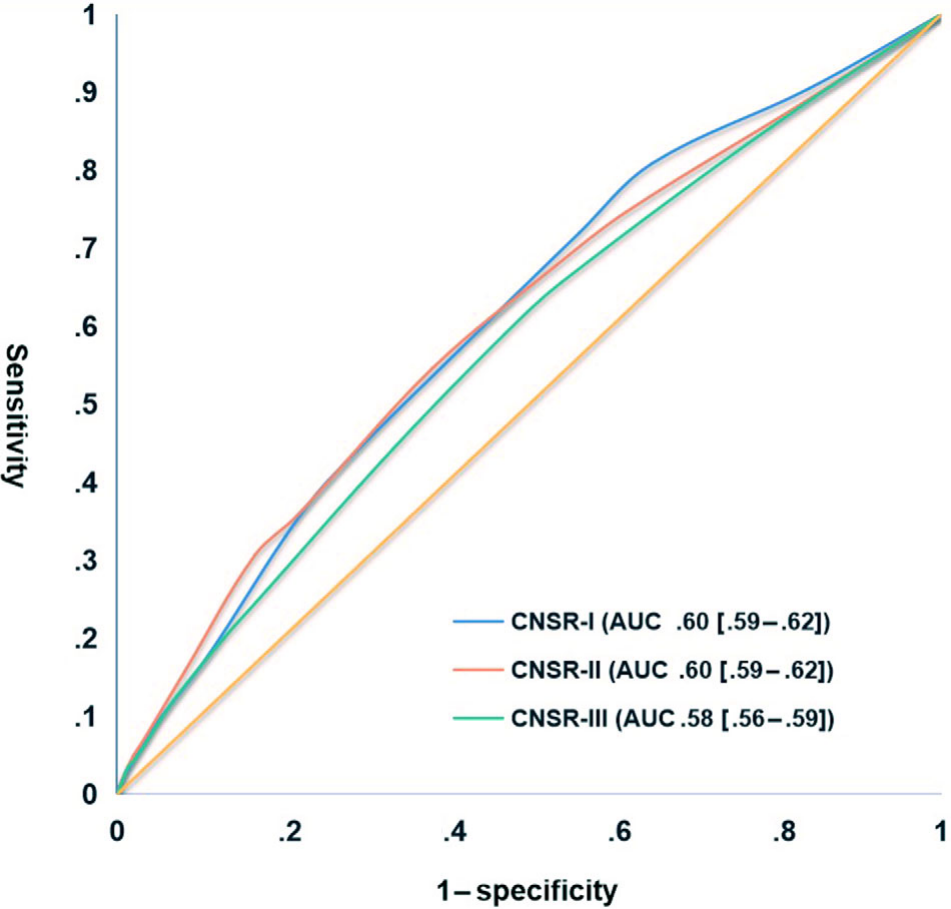
The trends of SPI‐II for diagnosing 1‐year subsequent stroke in the CNSR‐I, CNSR‐II, and CNSR‐III. SPI‐II, Stroke Prognosis Instrument‐II; CNSR, China National Stroke Registry.

**FIGURE 4:**
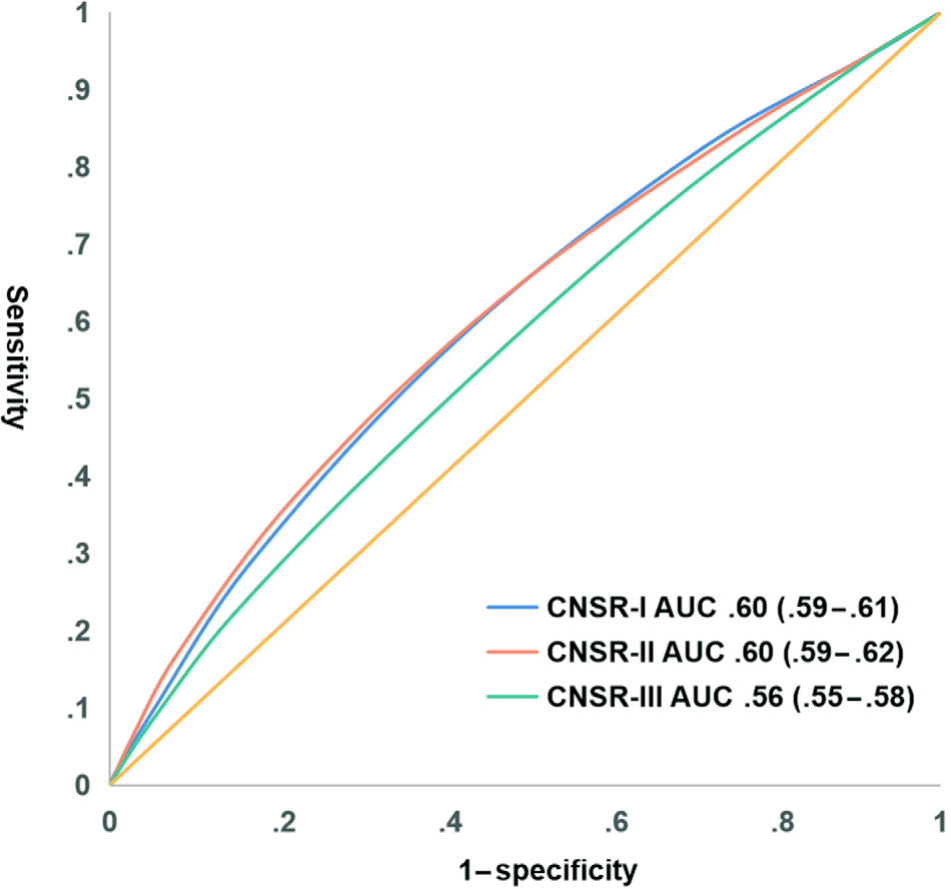
The trends of ESRS for diagnosing 1‐year subsequent stroke in the CNSR‐I, CNSR‐II, and CNSR‐III. ESRS, Essen Stroke Risk Score; CNSR, China National Stroke Registry.

The prediction power of SPI‐II and ESRS in CNSR‐I, CNSR‐II, and CNSR‐III is shown in Figures 3 and 4, separately. For SPI‐II, the AUC decreased from .60 (95% CI: .59–.62) in CNSR‐I to .60 (95% CI: .59–.62) in CNSR‐II and to .58 (95% CI: .56–.59) in CNSR‐III during the past 13 years. The declining trend was also found in the ESRS scale (CNSR‐I: .60 [95% CI: .59–.61]; CNSR‐II: .60 [95% CI: .59–.62]; and CNSR‐III: .56 [95% CI: .55–.58]). In addition, the predictive values of SPI‐II and ESRS for subsequent stroke at 1 year were similar between CNSR‐I and CNSR‐II (SPI‐II: CNSR‐I vs. CNSR‐II *p* = .66; ESRS: CNSR‐I vs. CNSR‐II *p* = .86). However, the prediction power of SPI‐II and ESRS in CNSR‐III was significantly lower than that in CNSR‐I and CNSR‐II (SPI‐II: CNSR‐I vs. CNSR‐III *p* = .009; CNSR‐II vs. CNSR‐III *p* = .03; ESRS: CNSR‐I vs. CNSR‐III *p* < .001; CNSR‐II vs. CNSR‐III *p* < .001).

## 4 DISCUSSION

In this pooled analysis of CNSR‐I, CNSR‐II, and CNSR‐III, we observed a modest performance of ESRS and SPI‐II for predicting subsequent stroke at 1 year in patients with AIS or TIA in China and that SPI‐II had a better performance than ESRS. Moreover, the two scales’ prediction power gradually decreased over the 13‐year study period.

In our study, the overall subsequent stroke risk for patients 1 year after their qualifying event was 10.7%, which is comparable with other studies (Lin et al., 2021). Recently, a meta‐analysis involving 1,075,014 stroke patients showed that the cumulative risk of subsequent stroke at 1 year was 10.4% (Tsao et al., 2022). However, our study indicated that the incidence of subsequent stroke after ischemic events depicted a U‐shape curve from 17.7% in CNSR‐I to 7.3% in CNSR‐II and to 9.7% in CNSR‐III. During the past 13 years, the secondary prevention strategies for AIS patients made substantial progress with more patients receiving effective treatment, including dual antiplatelet therapy for minor stroke (Wang et al., 2013), better control of hypertension, hyperlipidemia, and diabetes mellitus (Chimowitz et al., 2011). The lowest rate of subsequent stroke occurred in CNSR II. The CNSR II was a national quality improvement study, which included six acute performance measures—(1) intravenous tissue‐type plasminogen activator in patients who arrive within 2 h after symptom onset and treated within 3 h, (2) antithrombotic medication within 48 h of admission, (3) deep vein thrombosis prophylaxis within 48 h of admission for nonambulatory patients, (4) dysphagia screening before any oral intake, (5) carotid imaging, and (6) assessment or receiving of rehabilitation—and seven performance measures at discharge—(1) antithrombotic medication, (2) anticoagulation for atrial fibrillation, (3) antihypertensive medication for patients with hypertension, (4) medications for lowering low‐density lipoprotein ≥100 mg/dL, (5) hypoglycemia medication for diabetes mellitus, (6) smoking cessation, and (7) stroke education. At discharge, 85.5% of patients had smoking cessation, 90.3% of patients had antithrombotic therapy, and 97.7% of patients had stroke education; the lipid‐lowering drug, antihypertensive drug in hypertension patients, and glucose‐lowering drug rates in diabetes mellitus patients were 66.3%, 65.6%, and 74.0%, respectively. These may explain the U shape of subsequent stroke risk during the past 13 years in China.

Currently, various scales exist to assist clinicians in quantifying the short‐term or long‐term subsequent stroke risk in patients with AIS or TIA. The ESRS and SPI‐II are two of the most widely used scores for the prediction of long‐term risk of subsequent stroke (Chaudhary et al., 2019). Our study showed that SPI‐II performed better than ESRS, while a previous study analyzing the data from CNSR‐I showed ESRS and SPI‐II had similar predictive accuracy for subsequent stroke at 1 year because the study excluded 1381 patients with a history of atrial fibrillation in CNSR‐I (Meng et al., 2011). Many studies on the predictive power of SPI‐II and ESRS with a c‐statistic less than .70 indicated that the two scores both had limited predictive value of stroke recurrence within 1 year, which was in line with our study (Chaudhary et al., 2019).

Numerous scales purport to predict stroke outcomes from baseline clinical features. In fact, AIS and its recurrence are a multifactorial process with a combination of genetic, environmental, and vascular factors (Chaudhary et al., 2019). There was still an 8.3% residual risk of 1‐year subsequent stroke even in patients with long‐term adherence to guideline‐based secondary stroke prevention, and inflammatory factors (interleukin‐6, high‐sensitive C‐reactive protein) and relevant intracranial artery stenosis were independent risk factors of the residual risk (Ji et al., 2013; Koton et al., 2020). Furthermore, imaging (Nam et al., 2017), stroke subtype (Amarenco et al., 2016), and other biomarkers such as low‐density lipoprotein (Amarenco et al., 2006), copeptin (Greisenegger et al., 2015), homocysteine (Shi et al., 2018), and serum asymmetric dimethylarginine (Qin et al., 2021) all have been confirmed to have an association with subsequent stroke risk. Advances in the management of ischemic events have influenced subsequent stroke rates and the predictive performance of scales. Hence, it is necessary to evaluate the value of the above indicators and regularly refine the clinical risk scores based on the accuracy of prediction and the utility of the scale. Although the relationship between genetic variants and stroke has been reported, few studies have focused on the impact of genetic variants on stroke prognosis (Neurology Working Group of the Cohorts for Heart & Aging Research in Genomic Epidemiology [CHARGE] Consortium tSGNS & the International Stroke Genetics Consortium [ISGC], 2016; Malik et al., 2018). We note that the ongoing whole genome sequencing and genome‐wide analyses in CNSR‐III could help to refine our understanding on the genetic contribution to stroke outcomes (Cheng et al., 2021).

Our study is the first pooled analysis on the 13‐year trends in risk scores’ predictive values for subsequent stroke in patients with acute ischemic events in the Chinese population. There were several limitations in this study. First, we combined ischemic stroke, intracerebral hemorrhage, and subarachnoid hemorrhage as a single primary endpoint due to the lack of subgroup information in the CNSR‐I. However, considering different mechanisms of different strokes, further studies are warranted to determine if the discriminatory power of the presented risk scores differs based on separating out the individual types of stroke. Second, the participating hospitals among CNSR‐I, CNSR‐II, and CNSR‐III were not identical. Although the CNSR cohorts encompassed most areas of China, the patient characteristics in these selected hospitals could be different from county‐level or grassroots‐level hospitals. Hence, the rates for subsequent stroke may be underestimated in the overall Chinese population. Third, given that we excluded patients lacking the information for SPI‐II and ESRS, a selection bias might exist. Additionally, because our study included exclusively Chinese patients, the generalizability of the results should be further validated in other ethnic populations.

## 5 CONCLUSIONS

The predictive power of SPI‐II and ESRS for 1‐year subsequent stroke was limited and gradually decreased during the 13 years studied; thus, the scales may not be useful for current clinical practice. Further risk scale derivation may be warranted with additional imaging and biomarkers.

## AUTHOR CONTRIBUTORS

**Yunyun Xiong**: Study design; statistics; manuscript draft. **Shang Wang**: Manuscript draft. **Zixiao Li**: Study design. **Marc Fisher**: Critical revision of the manuscript. **Liyuan Wang**: Manuscript draft. **Yong Jiang**: Study design; data management. **Xinying Huang**: Statistics. **Xing‐Quan Zhao**: Study design. **Xia Meng**: Data collection. **Yongjun Wang**: Study design; critical revision of the manuscript.

## CONFLICT OF INTEREST STATEMENT

The authors declare no conflicts of interest.

### PEER REVIEW

The peer review history for this article is available at https://publons.com/publon/10.1002/brb3.2962

## Supporting information

Table S1 Comparison of CNSR series registries
Table S2 Parameters in SPI‐II and ESRS

## Data Availability

Data are available upon reasonable request from the corresponding author.
