# Supplementary material for: Thirteen‐year trends in risk scores predictive values for subsequent stroke in patients with acute ischemic event
Source: Brain Behav. 2023 Mar 28;13(5):e2962. doi: 10.1002/brb3.2962 (PMC10176011; doi:10.1002/brb3.2962)
Supplement: Supplementary file 1 — Table S1 Comparison of CNSR series registries Table S2 Parameters in SPI‐II and ESRS [file BRB3-13-e2962-s001.pdf]

Thirteen-year Trends in Risk Scores Predictive Values for Subsequent Stroke in Patients with Acute Ischemic Event

Yunyun Xiong, MD, PhD[1,2,3]; Shang Wang, MD, PhD[4]; Zixiao Li, MD, PhD[1,2,3,5]; Marc Fisher, MD[6]; Liyuan Wang, MD[1]; Yong Jiang, PhD[2]; Xinying Huang, MD, MSc[2]; Xing-Quan Zhao, MD, PhD[1,2]; Xia Meng, MD, PhD[2]; Yongjun Wang, MD, PhD[1,2,5,7]

[1]Department of Neurology, Beijing Tiantan Hospital, Capital Medical University, Beijing, China

[2]China National Clinical Research Center for Neurological Diseases, Beijing, China

[3]Chinese Institute for Brain Research, China

[4]Neurocardiology Center, Department of Neurology, Beijing Tiantan Hospital, Capital Medical University, Beijing, China

[5]National Center for Healthcare Quality Management in Neurological Diseases

[6]Department of Neurology, Stroke Division, Beth Israel Deaconess Medical Center, Harvard Medical School, Boston, MA

[7]Advanced Innovation Center for Human Brain Protection, Capital Medical University

Running title: Trends of scales for stroke recurrence prediction

Word count:2 416

Please correspondence to: Yongjun Wang, Department of Neurology, Beijing Tiantan Hospital, Capital Medical University, Beijing, China. Email: yongjunwang@ncrcnd.org.cn

Acknowledgement None.

Contributors Yunyun Xiong: study design, statistics and manuscript draft; Shang Wang: manuscript draft; Zixiao Li: study design; Marc Fisher: critical revision of the manuscript; Liyuan Wang: manuscript draft; Yong Jiang: study design and data management; Xinying Huang: statistics; Xing-Quan Zhao: study design; Xia Meng: data collection; Yongjun Wang: study design and critical revision of the manuscript.

Funding The project was supported by the Capital's Funds for Health Improvement and Research (2020-1-2041), National Natural Science Foundation of China (81870905, 82171272), Beijing Municipal Science and Technology Commission (Z211100003521019) and Beijing Hospitals Authority (PX2022019).

Competing interests None declared.

Patient consent for publication Not required.

Ethics approval All procedures performed in studies involving human participants were in accordance with the ethical standards of the institutional research committee and the principles of the Declaration of Helsinki. The study was approved by the Institutional Review Board of the Beijing Tiantan Hospital (IRB approval number: KY2015-001-01). Participants gave informed consent before taking part.

Data availability statement Data are available upon reasonable request from the

corresponding author.

Table S1 Comparison of CNSR series registries

Characteristics	CNSR-I<br>2007-2008<br>(N=21,902)	CNSR-II<br>2012-2013<br>(N=25,018)	CNSR-III<br>2015-2018<br>(N=15,166)
Purpose	To evaluate the quality of care for stroke patients in China	To assess the change in the quality of stroke care	To evaluate imaging and biomarker prognostic determinants of stroke
Participating hospitals	132 sites from 27 provinces and four municipalities, including 100 grade III and 32 grade II urban hospitals	219 sites (72 hospitals participating in both CNSR phase 1 and 2, 59 sites only in CNSR phase 1 and 147 hospitals only in phase 2)	201 sites from 22 provinces and four municipalities, including 163 grade III and 38 grade II urban hospitals
Onset to admission (days)	≤14	≤7	≤7
Target population	Patients with ischaemic stroke,	Similar to the CNSR-I but only	Patients with ischaemic stroke or TIA

	hemorrhagic stroke or TIA	focused on ischaemic stroke and TIA	
Genetic, imaging and biological sample collection	No	No	Yes
Follow-up (years)	1	1	5
Follow-up data collection	Over the telephone at three, six, 12, 18, and 24 months	Over the telephone	Face-to-face interview at 3 months and over the telephone at 6 months and 1-5 year annually.

CNSR, China National Stroke Registry; TIA, transient ischemic attack.

Table S2 Parameters in SPI-II and ESRS

Score	SPI-Ⅱ	ESRS
Risk factors (points)	Age > 70 years (2)<br>Diabetes mellitus (3)<br>Hypertension (1)<br>Coronary heart disease (1)<br>Distinction between stroke and TIA at baseline event (2)<br>Congestive heart failure (3)<br>Prior stroke (3)	Age<br>< 65 years (0)<br>65-75 years (1)<br>>75 years (2)<br>Hypertension (1)<br>Diabetes mellitus (1)<br>Previous myocardial infarction (1)<br>Other cardiovascular disease (except myocardial infarction and atrial fibrillation) (1)<br>Peripheral artery disease (1)<br>Smoking (1)<br>Additional TIA or ischemic stroke in addition to a qualifying event (1)
Risk groups	Low-risk: 0-3 points<br>Medium-risk: 4-7 points<br>High-risk: 8-15 points	Low-risk group: (0-2 points)<br>High-risk group: (⩾3 points)

SPI-II, Stroke Prognosis Instrument-II; ESRS, Essen Stroke Risk Score; TIA, transient ischemic attack.
